# The expression of the adenosine pathway markers CD39 and CD73 in salivary gland carcinomas harbors the potential for novel immune checkpoint inhibition

**DOI:** 10.1007/s00432-022-04211-x

**Published:** 2022-07-28

**Authors:** Arthur Bauer, Niklas Gebauer, Juliana Knief, Lars Tharun, Nele Arnold, Armin Riecke, Konrad Steinestel, Hanno M. Witte

**Affiliations:** 1 Department of Hematology and Oncology, Federal Armed Forces Hospital Ulm, Oberer Eselsberg 40, 89081 Ulm, Germany; 2 Institute of Pathology and Molecular Pathology, Federal Armed Forces Hospital Ulm, Oberer Eselsberg 40, 89081 Ulm, Germany; 3 Department of Hematology and Oncology, University Hospital of Schleswig-Holstein, Campus Luebeck, Ratzeburger Allee 160, 23538 Lübeck, Germany; 4 Institute of Pathology, Marienkrankenhaus Hamburg, Alfredstraße 9, 22087 Hamburg, Germany; 5 Institute of Pathology, University Hospital of Schleswig-Holstein, Campus Luebeck, Ratzeburger Allee 160, 23538 Lübeck, Germany; 6 Department of ENT, Federal Armed Forces Hospital Hamburg, Lesserstraße 180, 22049 Hamburg, Germany

**Keywords:** SGC, CD39, CD73, Adenosine signaling, Immunotherapy

## Abstract

**Background:**

In salivary gland carcinomas (SGC), there is only a small fraction of entities that appears to profit from immune checkpoint inhibition (ICI). Recent findings connected the activation of adenosine-signaling with a tolerogenic microenvironment. Therefore, the inhibition of adenosine pathway markers (CD39 and/or CD73) can augment ICI and/or display a novel immunotherapeutic strategy beyond ICI. Here, we assessed the immuno-histochemical expression of CD39 and CD73 across a wide spectrum of SGCs.

**Methods:**

In total, 114 patients with SGCs consecutively diagnosed between 2001 and 2021 were assessed for clinicopathological baseline characteristics and underwent confirmatory histopathological review. Immunohistochemical expression levels of CD39 and CD73 were assessed by applying the tumor proportion score (TPS) and the immune proportional score (IPS) comparable to PD-L1 expression analysis in routine clinical practice. Additionally, findings were correlated with PD-L1 expression levels.

**Results:**

The median age was 60.6 and 51.8% patients were female. The cohort covered a spectrum of eight distinct entities. Advanced-stage disease (UICC/AJCC III/IVA-IVC) at initial diagnosis was present in the majority of patients (64/114). Immunohistochemical staining revealed positivity for CD39 and CD73 in 48.2% and 21.1% on tumor cells (TPS ≥ 1%) as well as 46.4% and 42.9% within the immune cell infiltrate (IPS ≥ 1%), respectively. Further comparative analyses revealed immune-cold entities such adenoid cystic carcinoma (AdCC), immune-hot tumors such as adenocarcinoma, not otherwise specified (AC (NOS)) and entities with intermediate immunologic features such as acinic cell carcinoma (ACC).

**Conclusion:**

Current results indicate entity-specific adenosine signaling signatures. These findings suggest that the adenosine pathway plays a decisive role in tumor immunity among the major spectrum of SGCs. Targeting the adenosine pathway might pose a promising therapeutic option for selected entities.

**Supplementary Information:**

The online version contains supplementary material available at 10.1007/s00432-022-04211-x.

## Introduction

Salivary gland carcinomas (SGC) are rare malignancies (3–6% of all head and neck cancers). The current version of the World Health Organization (WHO) classification distinguishes 20 individual entities of SGC (El-Naggar et al. 2017). That implies that SGCs show biological heterogeneity (e.g., metastatic pattern, growth rate) depending on the respective histological subtype (Witte et al. 2022; Katabi and Lewis 2017). Due to the rarity and the biological diversity of these tumors, novel therapeutic strategies beyond surgical debulking, radiation therapy and/or conventional poly-chemotherapy are insufficiently assessed (Creagan et al. 1988; Katabi et al. 2014; Laurie et al. 2011). Especially in advanced-stage disease, the identification of promising novel therapeutic targets remains sparsely characterized (Witte et al. 2022).

In recent years, there is growing evidence for the efficacy of immunotherapeutic approaches such as checkpoint inhibition (ICI) across the spectrum of several solid tumor entities (Balar et al. 2017; Reck et al. 2019). Moreover, PD-L1 expression has emerged as a reliable biomarker of therapeutic relevance in large subset of solid tumors. However, for the majority of SGC subtypes, ICI was found to be ineffective (Fayette et al. 2019; Cohen et al. 2018). Since PD-L1 expression levels have been largely characterized across a variety of SGCs, the investigation of promising immunotherapeutic targets beyond PD-L1, such as the adenosine pathway markers CD39 [ecto-nuceloside triphosphate diphosphohydrolase-1 (ENTPD-1)] and CD73 (5′-nucleotidase), is still pending (Witte et al. 2020). The transmembrane enzymes CD39 and CD73 represent key regulators of the adenosine pathway (Allard et al. 2020a). The activation of CD39 and CD73 leads to the hydrolysis of extracellular ATP which serves as a ‘find me’ signal for immune cells resulting in an increase of immunosuppressive extracellular adenosine (Elliott et al. 2009). Adenosine-mediated immunosuppression confers chemo-resistance (Ferretti et al. 2019; Perrot et al. 2019). However, preclinical therapeutic blockade of the adenosine key regulators CD39 and CD73 can augment established ICI (PD-1/PD-L1/CTLA4), targeted therapeutic approaches (BRAF/MEK) and can overcome chemo-resistance (Allard et al. 2017, 2020b). Across a variety of malignancies, CD39 and CD73 expression levels have been analyzed and identified as a promising immunotherapeutic target (Ranjbar et al. 2019; Li et al. 2017). Ongoing basket studies investigate the efficacy of CD39 and/or CD73 blockade alone or in combination with ICI. Recent results (COAST trial) revealed promising efficacy for the combination of monoclonal anti-CD73 antibody oleclumab with PD-L1 checkpoint inhibitor durvalumab as a consolidation therapy following chemo-radiotherapy in stage III unresectable non-small cell lung cancer (NSCLC) resulting in an increased overall response rate (ORR: 30% vs. 17.9% with durvalumab alone) and 12-month progression-free survival (PFS) rate (62.6% vs. 33.9% with durvalumab alone) (Herbst et al. 2022). Ciforadenant was the adenosine pathway inhibitor for which first promising clinical results were presented (Fong et al. 2020). Anti-CD39 antibodies are currently undergoing early clinical evaluation and first results are eagerly awaited. Apart from a single study that investigated CD73 expression levels in acinic cell carcinomas (ACC) and mucoepidermoid carcinoma (MEC), the present study is the first to comprehensively analyze CD39 and CD73 expression levels across a large spectrum of SGCs to identify promising subtypes for adenosine pathway inhibition, especially for those entities in which the blockade of PD-1/PD-L1 was shown to be ineffective (Cohen et al. 2018; Ranjbar et al. 2019). In a relevant subset of cases, these investigations were interpreted alongside the context of immune cell infiltration and PD-L1 checkpoint expression levels. The current results indicate distinct, entity-specific CD39/CD73 expression profiles that contribute to future precision immunotherapeutic treatment guidance in selected SGC entities.

## Materials and methods

### Patients and samples

In this retrospective, multicenter study, institutional databases were reviewed to identify patients with SGCs, whose biopsy specimens (formalin-fixed paraffin-embedded; FFPE) from initial diagnosis had been referred to one of the participating institutions between 2001 and 2021. Patients with insufficient follow-up or with insufficient or unrepresentative tissue quality were excluded. Clinical information was collected from the original electronic patient files. Patient’s Eastern Cooperative Oncology Group (ECOG) performance status, staging data, treatment modalities and related response rates, pattern of relapse as well as information on survival were all anonymously coded alongside pathological assessment. The presence of disease progression within 6 months after initial diagnosis was defined as refractory disease. In contrast, relapse events were defined as the occurrence of disease later than 6 months after initial diagnosis. In total, 114 SGC patients complied with mandatory criteria for their inclusion in the current study.

### Histopathological evaluation

First, biopsy material and conventional slides relevant to the present study were sent to the Institute of Pathology and Molecular Pathology at Bundeswehrkrankenhaus Ulm (certified Center for Head and Neck Cancer) for centralized histopathological reevaluation and further immuno-histochemical investigations. Second, all diagnoses were reevaluated according to the 4th Edition of the World Health Organization (WHO) classification of head and neck tumors (El-Naggar et al. 2017).

### Immunohistochemistry

Immunohistochemistry was used to detect the expression of CD39 (HPA014067, Sigma Aldrich, dilution 1:100) and CD73 (HPA017357, Sigma Aldrich, dilution 1:500) on tumor cells and/or immune cells. As part of establishment of both immuno-histochemical markers, several solutions were tested for optimization. Concurrently, both positive and negative controls passed immuno-histochemical investigations for comparison purposes. In a relevant subset of cases, PD-L1 expression status and scoring (*n* = 69), the quantification of inflammatory cells (T and B cells in 69 cases) and the expression of CD117 (*n* = 95) were investigated as previously published (Witte et al. 2020). After deparaffinization (EZ-Prep^®^) and rehydration (ethanol), the sections (4 μm in thickness) were preconditioned with cell conditioning buffer 1 (CC1; 32 min) for antigen retrieval and then mixed with primary antibodies as part of a first incubation period of 20 min. Subsequently, washing and a second incubation period of 32 min with biotinylated secondary antibodies were conducted. The conversion of the substrate 3-amino-9-ethylcarbazole (Ventana OptiView DAB IHC detection kit, Ref: 760-700, Mannheim, Germany) conduced to visualization of the immunoreaction. The quantification of inflammatory cells has been performed by manually counting CD3^+^ T lymphocytes and CD20^+^ B lymphocytes in three representative high-power fields (HPF). Afterward, a mean score for each case has been calculated. Immunohistochemical evaluation has been performed by two independent pathologists. In case of discrepant results, a conclusive consensus assessment was accomplished. Antibodies and positivity cut-offs employed in the current study are summarized in Supplementary Table S1. All antibodies are intended for in vitro diagnostic use and were employed following the manufacturer’s protocol on a Ventana Benchmark Ultra immunostainer (Roche, Mannheim, Germany). Tonsil tissue of normal (human) served as a positive control for all antibodies.

### CD39, CD73 and PD-L1 scoring

Percentual expression levels of CD39 and CD73 have been evaluated in tumor cells and immune cells using conventional microscopy. Positive cells on whole slides of tumor tissue were quantified by two experienced pathologists from our certified Head and Neck Cancer Center and then a consensus scoring was determined. Referring to PD-L1 scoring proposed by Schildhaus et al., the immune proportional score (IPS) defined as the percentual of immune cells showing any membrane expression of CD39^+^ or CD73^+^ (four subgroups: 0 =  < 1%; 1 = 1–5%; 2 = 5–10%; 3 =  > 10%) was calculated for each case. By analogy, the tumor proportional score (TPS) was defined as the percentual of viable tumor cells showing any membrane expression of CD39 or CD73 (Schildhaus 2018). Additionally, we performed PD-L1 scoring (TPS and immune cell (IC) score) in a relevant subset of cases (*n* = 69) in concordance with Schildhaus et al*.* as previously described in SGC (Witte et al. 2020).

### Treatment and outcome

Staging was performed in accordance with the 8th edition of the TNM and UICC/AJCC staging system for head and neck cancer (Huang and O'Sullivan 2017). Individual treatment decision-making was conducted in a multidisciplinary tumor board setting wherever available or in accordance with the treating physician’s choice. Treatment response was defined in keeping with the ‘response evaluation criteria in solid tumors’ (RECIST) of complete response (CR) and partial response (PR) (Eisenhauer et al. 2009). Overall (OS) and PFS were calculated from the date of initial diagnosis. The assessment of treatment-related toxicities was conducted in conformity with the National Cancer Institute Common Toxicity Criteria (NCI CTC, version 5.0) wherever sufficient documentation was available.

### Ethics statement

This retrospective study was approved by the ethics committee of the University of Ulm (Reference Nos. 488-18 and 416-21) and conducted in accordance with the Declaration of Helsinki. All tissue samples were collected for histologic examination and diagnosis purpose and anonymized for the use in this study. Therefore, informed consent was not required.

### Statistics

All statistical investigations were conducted using GraphPad PRISM 9 (San Diego, CA, USA), R-Studio v. 3.6.1 (Boston, MA, USA) and SPSS 26 (IBM, Armonk, NY, USA). Survival (OS and PFS) was estimated by means of the Kaplan–Meier method and univariate log-rank test. The Fisher’s exact test was used to analyze differences between categorial variables. Differences between continuous variables were analyzed using ANOVA and Tukey's multiple comparisons test. A *p* < 0.05 was regarded as statistically significant. Additionally, BioRender (Toronto, Canada) was used as a tool for visualization.

## Results

### Clinicopathological characteristics

**Fig. 1:**
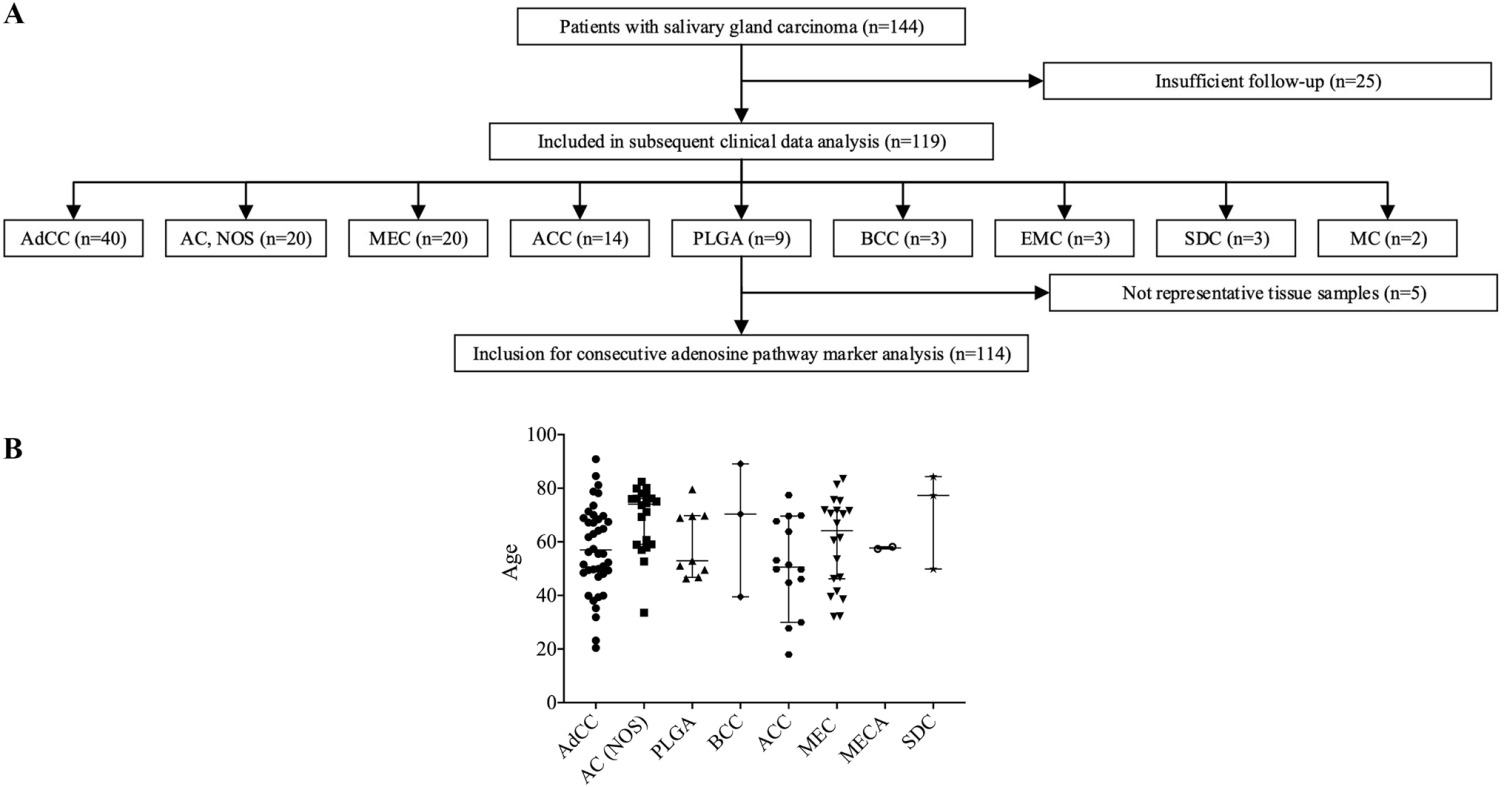
Flowchart depicting the composition of SGC patients included in the present study (**a**). Scatter plot visualizing the distribution of age for each SGC entity (**b**)

In total, 114 SGC patients were retrospectively enrolled in this multicenter trial. The composition of the study group is outlined in Fig. 1. In our cohort, the adenoid-cystic carcinoma (AdCC; 35.1%), the adenocarcinoma (not otherwise specified; AC (NOS); 17.5%), the mucoepidermoid carcinoma (MEC; 17.5%) and the acinic cell carcinoma (ACC; 12.3%) were the most frequent entities. Baseline clinicopathological characteristics are provided in Table 1. Median age was 60.6 years and gender distribution was equal in the overall study group. The majority of patients (76.8%) presented with good performance status (ECOG 0–1) and advanced-stage disease (UICC III/IV A-C; 56%). Only a minor subset of patients presented with primary metastatic disease (12%). However, especially, in patients with AC (NOS), metastatic disease (35%) and/or nodal disease (75%) was frequent at initial diagnosis.

**Table 1:** Baseline characteristics for all patients included in the study

Characteristic	Overall study group	AdCC	AC, NOS	MEC	ACC	PLGA	EMC	SGC	BCC	MC
	(*n* = 114)	(*n* = 40)	(*n* = 20)	(*n* = 20)	(*n* = 14)	(*n* = 9)	(*n* = 3)	(*n* = 3)	(*n* = 3)	(*n* = 2)
Male	55 (48.2%)	21 (52.5%)	10 (50.0%)	8 (40.0%)	7 (50.0%)	3 (33.3%)	1 (33.33%)	2 (66.7%)	2 (66.7%)	1 (50.0%)
Female	59 (51.8%)	19 (47.5%)	10 (50.0%)	12 (60.0%)	7 (50.0%)	6 (66.6%)	2 (66.7%)	1 (33.3%)	1 (33.3%)	1 (50.0%)
Median age	60.6	55.9	74.3	64.2	50.6	52.9	59.4	77.3	70.3	57.8
(Range, years)	(18.0–90.8)	(20.4–90.8)	(33.6–82.8)	(32.1–83.5)	(18.0–77.7)	(46.3–79.6)	(39.7–63.0)	(49.9–84.4)	(39.5–89.0)	(57.3–58.2)
BMI	26.3	26.9	25.5	26.6	26.4	28.4	23.2	26.3	29.4	23.9
(Median, range)	(18.7–45.7)	(18.7–45.7)	(20.8–45.5)	(21.3–35.0)	(22.2–34.9)	(19.5–41.7)	(22.6–25.2)	(23.7–39.9)	(23.9–42.7)	(21.5–26.3)
ECOG PS										
0–2	105 (92.1%)	38 (95.0%)	16 (88.9%)	18 (90.0%)	13 (92.9%)	9 (100.0%)	3 (100.0%)	3 (100.0%)	3 (100.0%)	2 (100.0%)
> 2	7 (7.9%)	2 (5.0%)	2 (11.1%)	2 (10.0%)	1 (7.1%)	–	–	–	–	–
CCI (Median, range)	4 (0–9)	4 (0–8)	6 (2–9)	4.5 (0–8)	3.5 (0–6)	2 (2–7)	3 (2–4)	7 (6–9)	5 (2–6)	3 (3)
LDH										
< 240 U/L	58 (67.4%)	21 (63.6%)	9 (56.3%)	14 (70.0%)	10 (83.3%)	3 (100.0%)	–	1 (50.0%)	–	–
> 240 U/L	28 (32.6%)	12 (36.4%)	7 (43.7%)	6 (30.0%)	2 (16.7%)	–	–	1 (50.0%)	–	–
B symptomes*										
Yes	8 (7.0%)	3 (7.5%)	3 (15.0%)	1 (5.0%)	–	–	–	–	1 (33.3%)	–
No	106 (93.0%)	37 (92.5%)	17 (85.0%)	19 (95.0%)	14 (100.0%)	9 (100.0%)	3 (100.0%)	3 (100.0%)	2 (66.7%)	2 (100.0%)
UICC/AJCC										
I	27 (23.7%)	10 (25.0%)	1 (5.0%)	7 (35.0%)	4 (28.6%)	2 (22.2%)	2 (66.7%)	–	1 (33.3%)	–
II	23 (20.2%)	6 (15.0%)	3 (15.0%)	3 (15.0%)	5 (35.7%)	2 (22.2%)	–	1 (33.3%)	1 (33.3%)	2 (100.0%)
III	21 (18.4%)	6 (15.0%)	2 (10.0%)	4 (20.0%)	4 (28.6%)	4 (44.4%)	–	1 (33.3%)	–	–
IVA	20 (17.5%)	8 (20.0%)	6 (30.0%)	4 (20.0%)	–	1 (11.1%)	1 (33.3%)	–	–	–
IVB	9 (7.9%)	6 (15.0%)	1 (5.0%)	–	1 (7.1%)	–	–	–	1 (33.3%)	–
IVC	14 (12.3%)	4 (10.0%)	7 (35.0%)	2 (10.0%)	–	–	–	1 (33.3%)	–	–
Primary localization										
GP	49 (43.0%)	8 (20.%)	13 (65.0%)	8 (40.0%)	14 (100.0%)	–	1 (33.3%)	3 (100.0%)	1 (33.3%)	1 (50.0%)
GSM	12 (10.5%)	6 (15.0%)	3 (15.0%)	2 (10.0%)	–	–	–	–	1 (33.3%)	–
GSL	6 (5.3%)	2 (5.0%)	–	3 (15.0%)	–	1 (11.1%)	–	–	–	–
P	26 (22.8%)	13 (32.5%)	–	3 (15.0%)	–	8 (88.9%)	2 (66.7%)	–	–	–
NC	3 (2.6%)	2 (5.0%)	1 (5.0%)	–	–	–	–	–	–	–
Others	18 (15.8%)	9 (22.5)	3 (15.0%)	4 (20.0%)	–	–	–	–	1 (33.3%)	1 (50.0%)
Nodal disease										
N0	78 (68.4%)	34 (85.0%)	5 (25.0%)	15 (75.0%)	10 (71.4%)	6 (66.7%)	3 (100.0%)	1 (33.3%)	2 (66.7%)	2 (100.0%)
N+	36 (31.6%)	6 (15.0%)	15 (75.0%)	5 (25.0%)	4 (28.6%)	3 (33.3%)	–	2 (66.7%)	1 (33.3%)	–
Metastatic disease										
M0	100 (87.7%)	36 (90.0%)	13 (65.0%)	18 (90.0%)	14 (100.0%)	9 (100.0%)	3 (100.0%)	2 (66.7%)	3 (100.0%)	2 (100.0%)
M +	14 (12.3%)	4 (10.0%)	7 (35.0%)	2 (10.0%)	–	–	–	1 (33.3%)	–	–
Second malignancy										
Yes	17 (14.9%)	3 (7.5%)	7 (35.0%)	1 (5.0%)	3 (21.4%)	1 (11.1%)	–	1 (33.3%)	–	1 (50.0%)
No	97 (85.1%)	37 (92.5%)	13 (65.0%)	19 (95.0%)	11 (78.6%)	8 (88.9%)	3 (100.0%)	2 (66.7%)	3 (100.0%)	1 (50.0%)

ACC, acinic cell carcinoma; AC (NOS), Adenocarcinoma; AdCC, Adenoid cystic carcinoma, AJCC, American Joint Committee on Cancer; BCC, Basal cell adenocarcinoma; BMI, Body-Mass-Index; CCI, Charlson Comorbidity Index; ECOG PS, Eastern Cooperative Oncology Group performance status; EMC, Epithelial myoepithelial carcinoma; GP, Gl; Parotis, GSL; Gl, Sublingualis; GSM, Gl, Submandibularis; LDH, lactate dehydrogenase; M, metastasis; MC, Myoepithelial carcinoma; MEC, Mucoepidermoid carcinoma; N, nodal metastases; NC, nasal cavity and paranasal sinuses; P, palate; PLGA, Polymorphous low-grade adenocarcinoma; SDC, Salivary duct carcinoma; UICC, Union for International Cancer Control

^*^Fever, night sweats and weight loss

### CD39/CD73 expression on tumor and immune cells

**Fig. 2:**
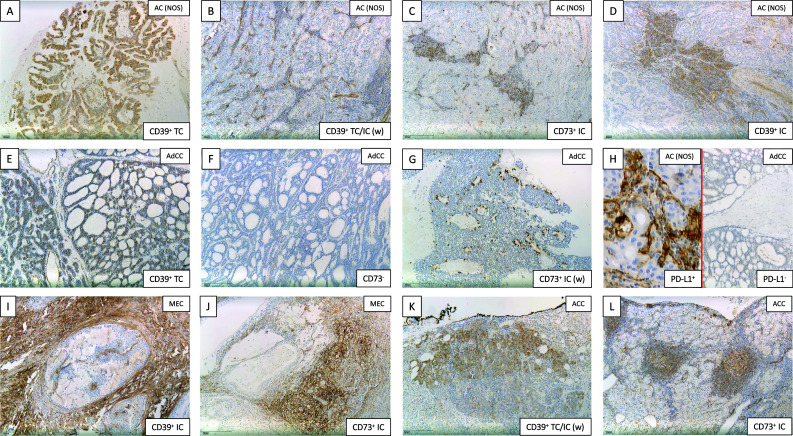
Representative immuno-histochemical staining for CD39 (**a**, **b**, **d**, **e**, **i**, **k**) and CD73 (**c**, **f**, **g**, **j**, **l**) as well as PD-L1 (**h**). This selection demonstrates adenosine marker expressions in AC (NOS) (**a**–**d**), AdCC (**e**–**g**), MEC (**i**, **j**) and ACC (**k**, **l**). We differentiated between expression on tumor cells (TC; **a**, **b**, **e**, **k**) and immune cells (IC; **b**, **c**, **d**, **e**, **g**, **i**, **j**, **k**, **l**)

Representative immuno-histochemical staining for the adenosine pathway markers CD39 and CD73 is demonstrated in Fig. 2. In 55 of 114 patients, immuno-histochemical assessment revealed CD39 positivity on tumor cells (TPS ≥ 1). Especially in AC (NOS) (55%) and polymorphous low-grade adenocarcinomas (PLGA) (78%), the majority of cases showed relevant CD39 expression on tumor cells. Moreover, a frequent CD39 expression on tumor cells was observed in rare entities included in the current cohort [3/3 epithelial–myoepithelial carcinoma (EMC), 3/3 salivary duct carcinoma (SDC), 3/3 basal cell adenocarcinoma (BCAC) and 2/2 myoepithelial carcinoma (MC)]. Immunohistochemical analysis of CD73 on tumor cells demonstrated lower expression levels compared to CD39 [4.4% (CD73) versus 12.5% (CD39) mean expression on tumor cells]. Regarding CD73 expression, only 24 of 114 SGC cases presented with a relevant TPS ≥ 1. Most frequent CD73 expression (TPS ≥ 1) on tumor cells was detected in MEC (30%), ACC (29%) and PLGA (33%).

**Fig. 3:**
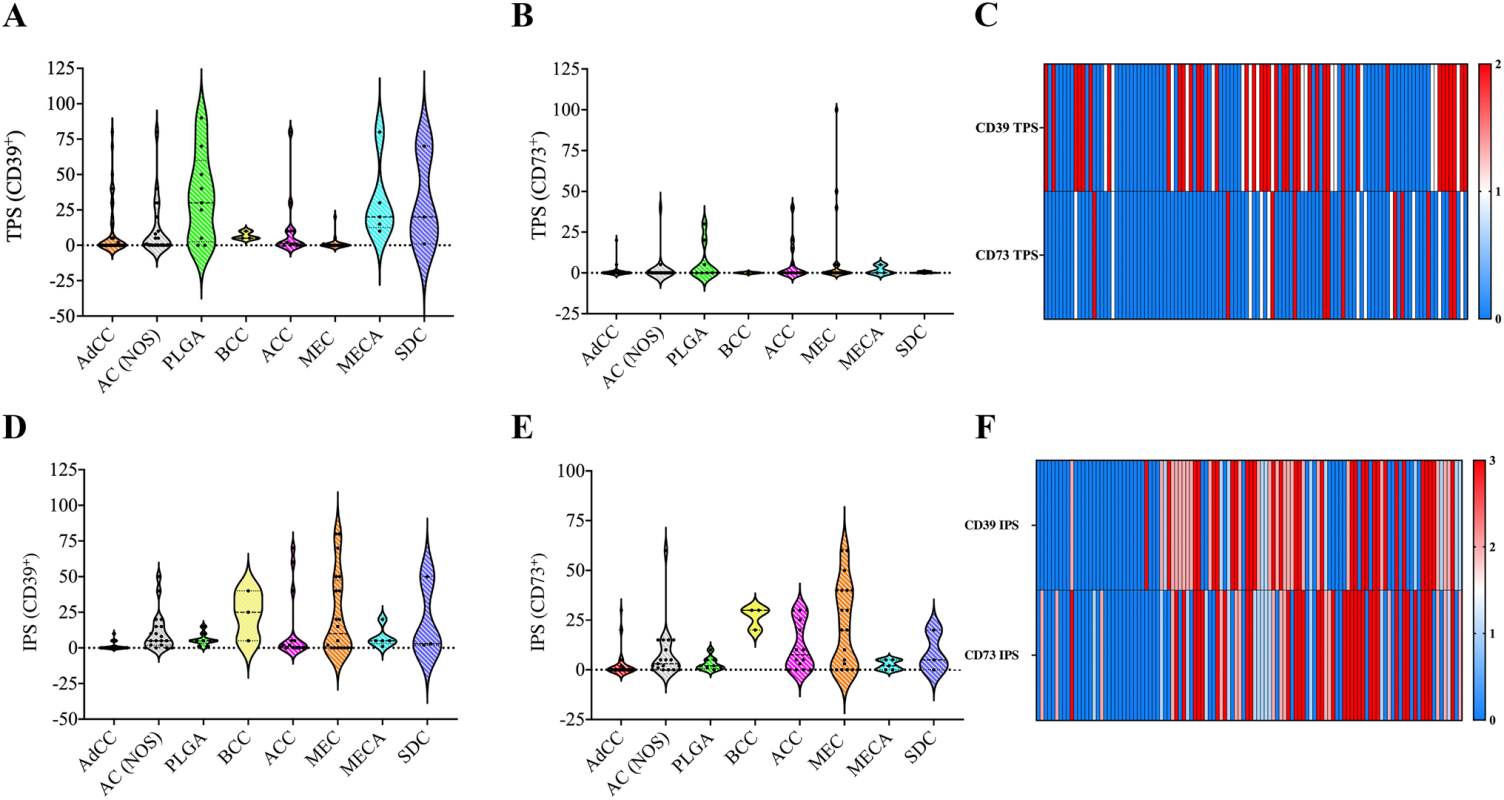
**a**, **b**, **d**, **e** Violin plots visualize relevant immuno-histochemical expression patterns of CD39 (**a**, **d**) and CD73 (**b**, **e**) on tumor cells (**a**, **b**) and immune cells (**d**, **e**) among several SGC entities. Additionally, heat maps were created to further visualize the composition of CD39 and CD79 expression on tumor cells (**c**) and immune cells (**f**) within the overall SGC study cohort

In the overall study group, expression levels for CD39 (53/114; mean 10%) and CD73 (49/114; mean 9%) on immune cells were comparable. The highest CD39 expression (IPS 2–3) was detected in the immune infiltrate of AC (NOS) (70%), PLGA (78%), MEC (55%) and rare entities included in the cohort, such as 2/3 EMC, 3/3 BCAC and 2/2 MC. A frequent CD73 expression on immune cells was observed in AC (NOS) (50%), MEC (65%) and ACC (64%) (rare entities: 2/3 SGC, 3/3 BCAC). The distribution of CD39 and/or CD73 expression levels on either tumor cells or immune cells is visualized in Fig. 3. Dedicated results from immuno-histochemical staining of adenosine pathway markers are summarized in Table 2.

**Table 2:** Immunohistochemical findings in the study cohort

Characteristics	Overall study group	AdCC	AC, NOS	MEC	ACC	PLGA	MECA	SDC	BCC	MC
	(*n* = 114)	(*n* = 40)	(*n* = 20)	(*n* = 20)	(*n* = 14)	(*n* = 9)	(*n* = 3)	(*n* = 3)	(*n* = 3)	(*n* = 2)
Male	55 (48.2%)	21 (52.5%)	10 (50.0%)	8 (40.0%)	7 (50.0%)	3 (33.3%)	1 (33.33%)	2 (66.7%)	2 (66.7%)	1 (50.0%)
Female	59 (51.8%)	19 (47.5%)	10 (50.0%)	12 (60.0%)	7 (50.0%)	6 (66.6%)	2 (66.7%)	1 (33.3%)	1 (33.3%)	1 (50.0%)
Ki-67 (median, range)	18%	20%	30%	5%	6%	9%	30%	25%	50%	40%
	(2–80%)	(8–80%)	(10–75%)	(2–15%)	(2–80%)	(2–30%)	(8–30%)	(15–60%)	(25–70%)	(30–50%)
TPS CD39 mean (range)	12.5%	11.2%	11.6%	1.3%	11.4%	34.4%	20%	30.3%	6.7%	47.5%
	(0–90%)	(0–80%)	(0–80%)	(0–20%)	(0–80%)	(0–90%)	(10–30%)	(1–70%)	(5–10%)	(15–80%)
TPS CD39 < 1	**59 (51.8%)**	**25 (62.5%)**	**9 (45.0%)**	**17 (85.0%)**	**6 (42.9%)**	2 (22.2%)	–	–	–	–
TPS CD39 1–5	16 (14.0%)	4 (10.0%)	3 (15.0%)	2 (10.0%)	3 (21.4%)	1 (11.1%)	–	1 (33.3%)	**2 (66.7%)**	–
TPS CD39 > 5	39 (34.2%)	11 (27.5%)	8 (40.0%)	1 (5.0%)	5 (35.7%)	**6 (66.7%)**	**3 (100.0%)**	**2 (66.7%)**	1 (33.3%)	**2 (100.0%)**
TPS CD73 mean (range)	4.4%	0.7%	2.3%	10.3%	5.7%	6.1%	26.7%	0.3%	0%	2.5%
	(0–100%)	(0–20%)	(0–40%)	(0–100%)	(0–40%)	(0–30%)	(0–60%)	(0–1%)		(0–5%)
TPS CD73 < 1	**90 (79.0%)**	**37 (92.5%)**	**16 (80.0%)**	**14 (70.0%)**	**10 (71.4%)**	**6 (66.7%)**	1 (33.3%)	**2 (66.7%)**	**3 (100.0%)**	1 (50.0%)
TPS CD73 1–5	12 (10.5%)	2 (5.0%)	3 (15.0%)	3 (15.0%)	1 (7.2%)	1 (11.1%)	–	1 (33.3%)	–	1 (50.0%)
TPS CD73 > 5	12 (10.5%)	1 (2.5%)	1 (5.0%)	3 (15.0%)	3 (21.4%)	2 (22.2%)	**2 (66.7%)**	–	–	–
IC CD39 mean (range)	10.1%	1.3%	11.9%	23.6%	13.1%	5.8%	3.7%	18.3%	23.3%	12.5%
	(0–80%)	(0–10%)	(0–50%)	(0–80%)	(0–60%)	(1–15%)	(1–5%)	(2–50%)	(5–40%)	(5–20%)
IPS CD39 0–1	**61 (53.5%)**	**32 (80.0%)**	6 (30.0%)	9 (45.0%)	**9 (64.3%)**	2 (22.2%)	1 (33.3%)	**2 (66.7%)**	–	–
IPS CD39 2–3	53 (46.5%)	8 (20.0%)	**14 (70.0%)**	**11 (55.0%)**	5 (35.7%)	**7 (77.8%)**	**2 (66.7%)**	1 (33.3%)	**3 (100.0%)**	**2 (100.0%)**
IPS CD73 mean (range)	8.9%	2.3%	8%	22.4%	12%	2.9%	2.3%	8.3%	26.7%	2.5%
	(0–60%)	(0–30%)	(0–60%)	(0–60%)	(0–30%)	(0–10%)	(0–5%)	(0–20%)	(20–30%)	(0–5%)
IPS CD73 0–1	**65 (57.0%)**	**33 (82.5%)**	10 (50.0%)	7 (35.0%)	5 (35.7%)	**6 (66.7%)**	**2 (66.7%)**	1 (33.3%)	–	1 (50.0%)
IPS CD73 2–3	49 (43.0%)	7 (17.5%)	10 (50.0%)	**13 (65.0%)**	**9 (64.3%)**	3 (33.3%)	1 (33.3%)	**2 (66.7%)**	**3 (100.0%)**	1 (50.0%)

Most frequent categories detected for a feature investigated in the present study were marked in bold for each entity

ACC, Acinic cell carcinoma; AC (NOS) Adenocarcinoma; AdCC, Adenoid cystic carcinoma; BCC, Basal cell adenocarcinoma; MECA, Epithelial myoepithelial carcinoma; IPS, immune proportional score; MC, Myoepithelial carcinoma; MEC, Mucoepidermoid carcinoma; PLGA, Polymorphous low-grade adenocarcinoma; SDC, Salivary duct carcinoma; TPS, tumor proportion score; (–), none

### Adenosine pathway signatures

**Fig. 4:**
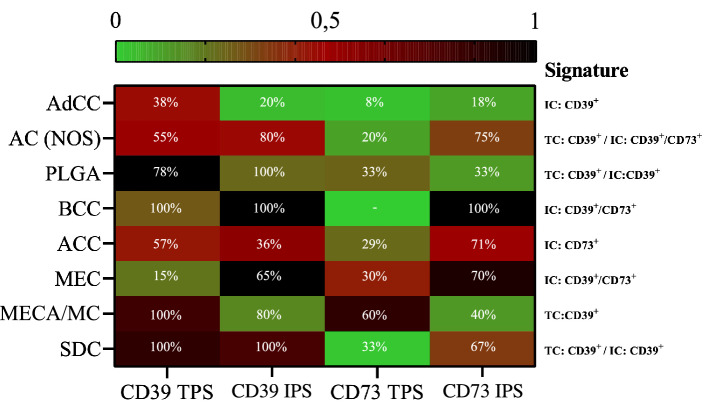
Heatmap (G) outlining distinct CD39/CD73 expression signatures and the percentual quantitative distribution of relevant expressions on tumor cells (TC) and/or immune cells (IC) for each SGC subgroup

As described above, the majority of entities included in the study presented with relevant expression patterns of adenosine pathway markers (CD39 and/or CD73). Looking at the entire study group, it can be concluded that compared to CD73 expression, the expression of CD39 on tumor cells is a more common feature in SGC (Fig. 3c). On immune cells, expression levels of CD39 and CD73 are comparable across the current SGC cohort. A specific adenosine pathway immune profile was detected for each entity. AdCC appeared with the coldest immune infiltrate regarding the expression of CD39 and/or CD73. However, a relevant subset of AdCC expressed CD39 on tumor cells but not on immune cells. As opposed to this, AC (NOS) had a hot adenosine pathway-related immune infiltrate with CD39 expression on tumor and immune cells as well as CD73 expression on immune cells harboring the potential for multiple targeted therapeutic options. Interestingly, in PLGA, CD73 expression remained sparse, whereas frequent CD39 expression levels were observed on immune and tumor cells. The same expression signature (CD39^+^ on tumor and immune cells) has been shown for SDC. The most prominent immuno-histochemical results in ACC were found for CD73 expression on tumor cells. MEC showed frequent expression of both adenosine pathway markers (CD39 and CD73) on immune cells but not on tumor cells. The composition of all entity-related adenosine marker expression signatures is depicted in Fig. 4.

### Analysis of the immune infiltrate

**Fig. 5:**
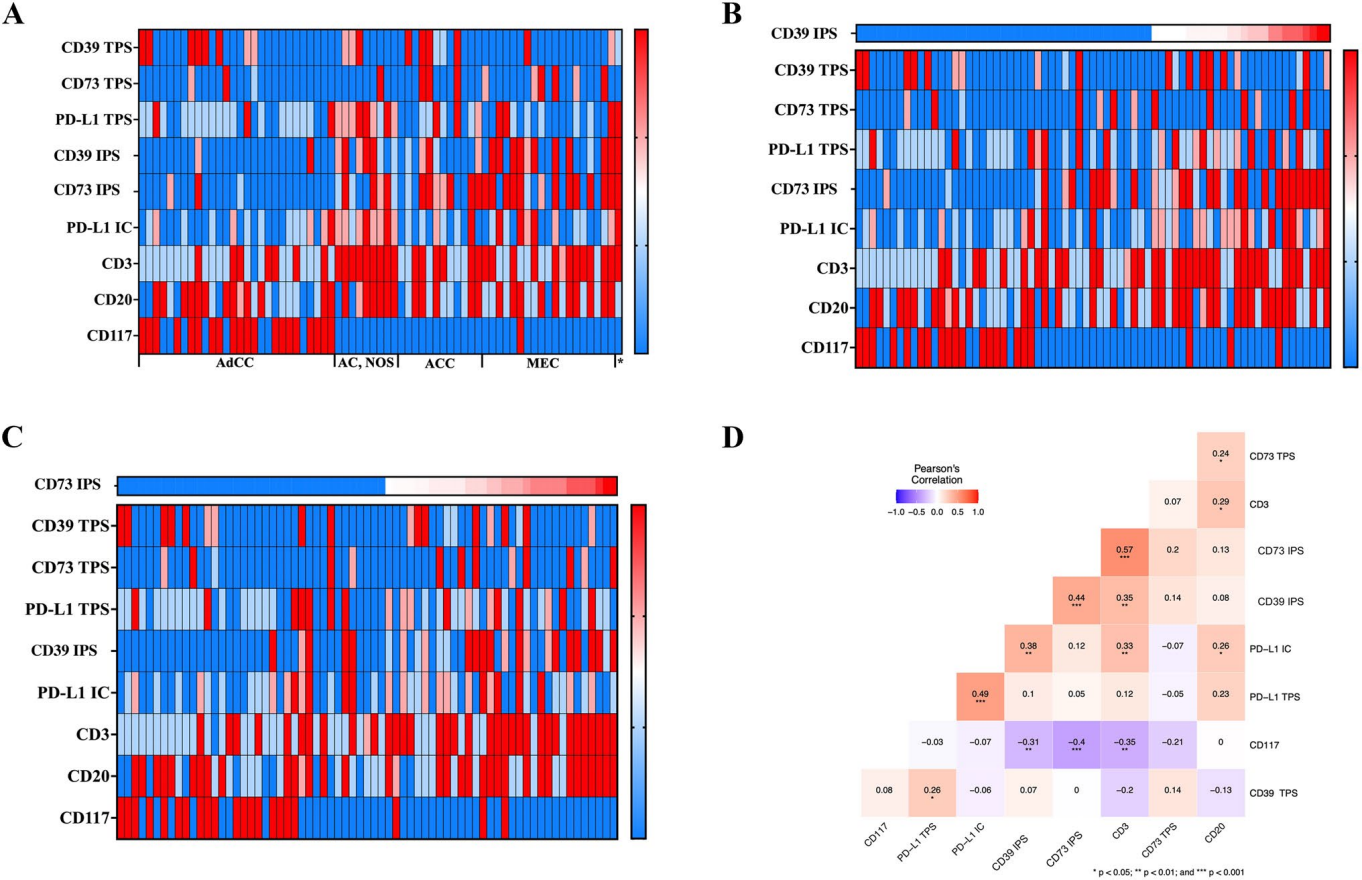
Heatmaps (**a**–**c**) demonstrating immuno-histochemical analysis of the immune checkpoints (CD39, CD73 and PD-L1) on tumor cells and immune cells, immune cell markers (CD3 and CD20) and KIT (CD117) among the spectrum of 69 available SGC cases. Rows represent immune (checkpoint) markers and columns denote samples (red, relatively higher expression level (CD39, CD73, PD-L1) or immune cell density; blue, lower expression level (CD39, CD73, PD-L1) or immune cell density). Visualization of Pearson’s correlation (**d**) with respect to immune checkpoint markers and immune cell infiltrate as well as CD117 expression levels. High degrees of correlation are colored in red and low degrees of correlation are colored in blue. Significant correlations are marked with *(*p* < 0.05), **(*p* < 0.01) and ***(*p* < 0.001). *Salivary duct carcinoma

Our data allowed an immuno-histochemical analysis of the immune infiltrate in a subset of 69 SGC cases (Supplementary Table S2). Here, a frequent PD-L1 expression was detectable in AC (NOS) on tumor and immune cells, respectively. In MEC, we detected PD-L1 expression on immune cells (Fig. 5a). Additionally, CD39^+^ immune cells showed high expression levels of PD-L1. In particular, immune cells showed co-expression of CD39 and PD-L1 (Fig. 5b). The infiltration of B cells (CD20) was heterogeneously distributed across the whole study group. In contrast, the density of tumor-infiltrating T cells (TIL; CD3) correlated with the expression of both adenosine pathway markers (CD39 and CD73) on immune cells (Fig. 5b–d). Consequently, only a small fraction of AdCC showed relevant TILs (Fig. 5a). In conformity with literature characteristic, CD117 expression (immuno-histochemical correlate of c-kit activation) was almost exclusively present in AdCC (Fig. 5a). Pearson’s correlation including the components of the immune infiltrate confirmed abovementioned heatmap interpretations (Fig. 5d). Especially the expression of CD73 on immune cells significantly correlated with the infiltration of T cells (CD3) (*r* = 0.57; *p* < 0.001). Moreover, there was a significant correlation regarding the expression of both adenosine pathway markers (CD39 and CD73) on immune cells (*r* = 0.44; *p* < 0.001) but not on tumor cells. The expression of CD117 negatively correlated with T-cell infiltration (*r* = − 0.35; *p* < 0.01) as well as CD39 (*r* = − 0.31; *p* < 0.01) and CD73 (*r* = − 0.4; *p* < 0.001) expression on immune cells. Interestingly, expression levels of PD-L1 on immune cells closely correlated with CD39 positivity on those cells (*r* = 0.38; *p* < 0.01). The same feature was observed on tumor cells (PD-L1 and CD39 co-expression), harboring the potential for augmentative immunotherapeutic combinations (*r* = 0.26; *p* < 0.05).

### Treatment and survival outcome

**Fig. 6:**
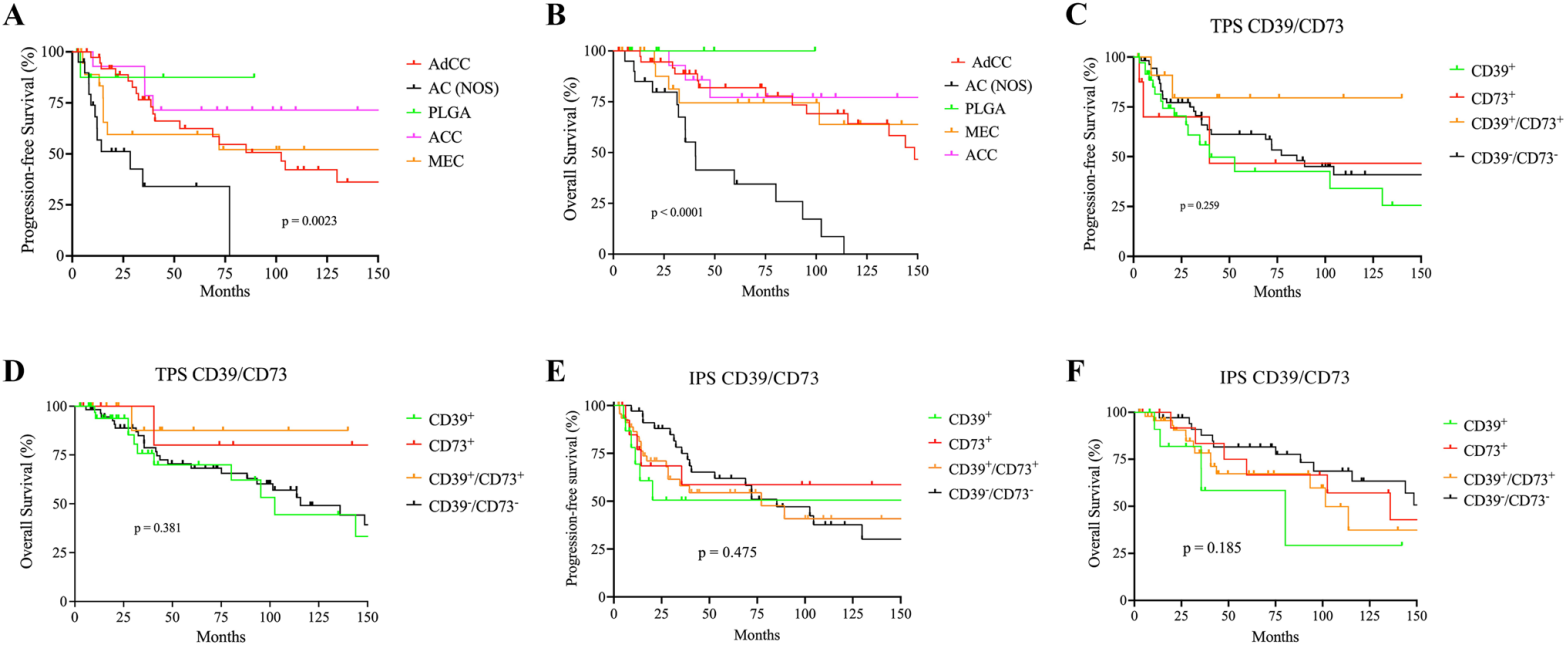
Comparative Kaplan–Meier survival analysis revealed AC (NOS) to be significantly associated with worst clinical prognosis regarding both PFS (**a**) and OS (**b**). Further, Kaplan–Meier analysis showed no significant impact on neither PFS (**c**, **e**) nor OS (**d**, **f**) based on CD39 and CD73 expression on tumor cells (**c**, **d**) or immune cells (**e**, **f**) in SGCs

Although the majority of cases presented with advanced-stage disease (56%), surgical resection with curative intent was performed in 107/114 (93.8%) cases after initial diagnosis. Radiotherapy was performed in 54/114 patients (47.3%). The addition of adjuvant radiotherapy led to an elevation of complete remissions (CR) in 30/48 cases (62.5%). Only ten patients underwent chemotherapy as first line treatment. Consecutively, CR was achieved in 81/114 cases (71%) and the overall response rate (ORR) was 97.4%. In total, 54 patients experienced relapse (37%) or refractory disease (7%). In relapse/refractory setting, 29/54 (54%) received systemic cyto-reductive treatment, such as chemotherapeutic, immuno- or targeted therapeutic approaches. Detailed treatment characteristics of the study cohort are outlined in Table 3. Median overall survival (OS) and progression-free survival (PFS) across all SGC entities were 42.3 months and 29.5 months, respectively. Upon comparative survival analysis, AC (NOS) was associated with the poorest prognosis (*p* < 0.0001, OS: 35.5 months; *p* = 0.0023, PFS: 13.2 months; Fig. 6a, b; Supplementary Table S3). Based on CD39 and CD73 expression on either tumor (TPS) or immune cells (IPS), Kaplan–Meier analysis revealed no significant survival benefit for any subgroup (Fig. 6c–f). There was a trend toward a favorable OS for SGC with CD73 expression on tumor cells, failing to achieve the level of significance (*p* = 0.381) (Fig. 6d). Noteworthy, no patient received a novel immunotherapeutic approach targeting the adenosine pathway and only a limited fraction of patients (*n* = 3) received ICI in a r/r setting. Across the entire study group, the toxicity profile was moderate and predominantly hematological in nature with nine cases of grade III/IV cytopenia.

**Table 3:** Treatment modalities of patients with salivary gland carcinoma included in the study

Characteristics	Overall study group	AdCC	AC, NOS	MEC	ACC	PLGA	EMC	SDC	BCC	MC
	(*n* = 114)	(*n* = 40)	(*n* = 20)	(*n* = 20)	(*n* = 14)	(*n* = 9)	(*n* = 3)	(*n* = 3)	(*n* = 3)	(*n* = 2)
*1st line therapy*										
Surgical resection	107 (93.9%)	36 (90.0%)	19 (95.0%)	20 (100.0%)	14 (100.0%)	9 (100.0%)	3 (100.0%)	2 (66.7%)	3 (100.0%)	1 (50.0%)
Radiotherapy	54 (47.3%)	22 (55.0%)	11 (55.0%)	10 (50.0%)	5 (35.7%)	2 (22.2%)	1 (33.3%)	1 (33.3%)	1 (33.3%)	1 (50.0%)
Chemotherapy (CTX)	10 (8.7%)	3 (7.5%)	3 (15.0%)	2 (10.0%)	1 (7.1%)	–	–	1 (33.3%)	–	–
CAP	7	3	2	1	1	–	–	–	–	–
Others	3	–	1	1	–	–	–	1	–	–
Best response 1st line										
CR	**81 (71.1%)**	**30 (75.0%)**	9 ((45.0%)	**15 (75.0%)**	**11 (78.6%)**	**9 (100.0%)**	**3 (100.0%)**	–	**3 (100.0%)**	1 (50.0%)
PR	28 (24.6%)	10 (25.0%)	**10 (50.0%)**	5 (5.0%)	2 (14.3%)	–	–	1 (33.3%)	–	–
SD	2 (1.7%)	–	1 (5.0%)	–	–	–	–	1 (33.3%)	–	–
PD	3 (2.6%)	–	–	–	1 (7.1%)	–	–	1 (33.3%)	–	1 (50.0%)
Lines of therapy (mean, range)	1.89	1.78	2.45	1.95	1.79	1.56	1	2.33	2	1
	(1–7)	(1–4)	(1–6)	(1–6)	(1–7)	(1–3)	(1)	(2–3)	(1–3)	(1)
Treatment of relapses										
Surgical resection	26 (22.8%)	13 (32.5%)	2 (10.0%)	6 (30.0%)	–	3 (33.3%)	–	–	1 (33.3%)	1 (50.0%)
Radiotherapy	16 (14.0%)	5 (12.5%)	8 (40.0%)	1 (5.0%)	1 (7.1%)	–	–	–	–	1 (50.0%)
Chemotherapy	18 (15.8%)	9 (22.5%)	5 (25.0%)	1 (5.0%)	2 (14.2%)	–	–	1 (33.3%)	–	–
CAP	6	3	2	–	1	–	–	–	–	–
MFP	4	3	–	1	–	–	–	–	–	–
Others	8	3	3	–	1	–	–	1	–	–
Targeted therapy	11 (9.6%)	7 (17.5%)	2 (10.0%)	–	1 (7.1%)	–	–	1 (33.3%)	–	–
mTor inhibition	3	1	1	–	–	–	–	1	–	–
EGFR inhibition	3	2	–	–	1	–	–	–	–	–
Immunotherapy	3	2	1	–	–	–	–	–	–	–
Others	2	2	–	–	–	–	–	–	–	–
CTX-associated toxicity profile										
Cytopenia grade III/IV	9 (50.0%)	3 (33.3%)	2 (40.0%)	1 (100.0%)	2 (100.0%)	–	–	–	–	–
Acute kidney disease	3 (16.7%)	3 (33.3%)	–	–	–	–	–	–	–	–
Sepsis	3 (16.7%)	1 (11.1%)	1 (20.0%)	1 (100.0%)	–	–	–	–	–	–
Cardiotoxicity	1 (5.6%)	1 (11.1%)	–	–	–	–	–	–	–	–
Surgical treatment-associated toxicity										
Neuronal damage	14 (13.1%)	9 (25.0%)	3 (15.8%)	–	2 (14.2%)	–	–	–	–	–
Fistula	2 (1.9%)	1 (2.8%)	–	1 (5.0%)	–	–	–	–	–	–
Others	11 (10.3%)	1 (2.8%)	2 (10.5%)	3 (15.0%)	2 (14.2%)	2 (66.7%)	1 (33.3%)	–	–	–

Most frequent categories detected for a feature investigated in the present study were marked in bold for each entity

ACC, Acinic cell carcinoma; AC, NOSAdenocarcinoma; AdCC, Adenoid cystic carcinoma; BCC, Basal cell adenocarcinoma; CAP, cisplatin/adriamycin/cyclophosphamid; CTX, Chemotherapy; EGFR, epidermal growth factor receptor; EMC, Epithelial–myoepithelial carcinoma; MC, Myoepithelial carcinoma; MEC, Mucoepidermoid carcinoma; MFP, methotrexat/5-fluoruracil/cisplatin; mTOR, mechanistic target of rapamycin; PLGA, Polymorphous low-grade adenocarcinoma; SDC, salivary duct carcinoma

### Perspectives

**Fig. 7:**
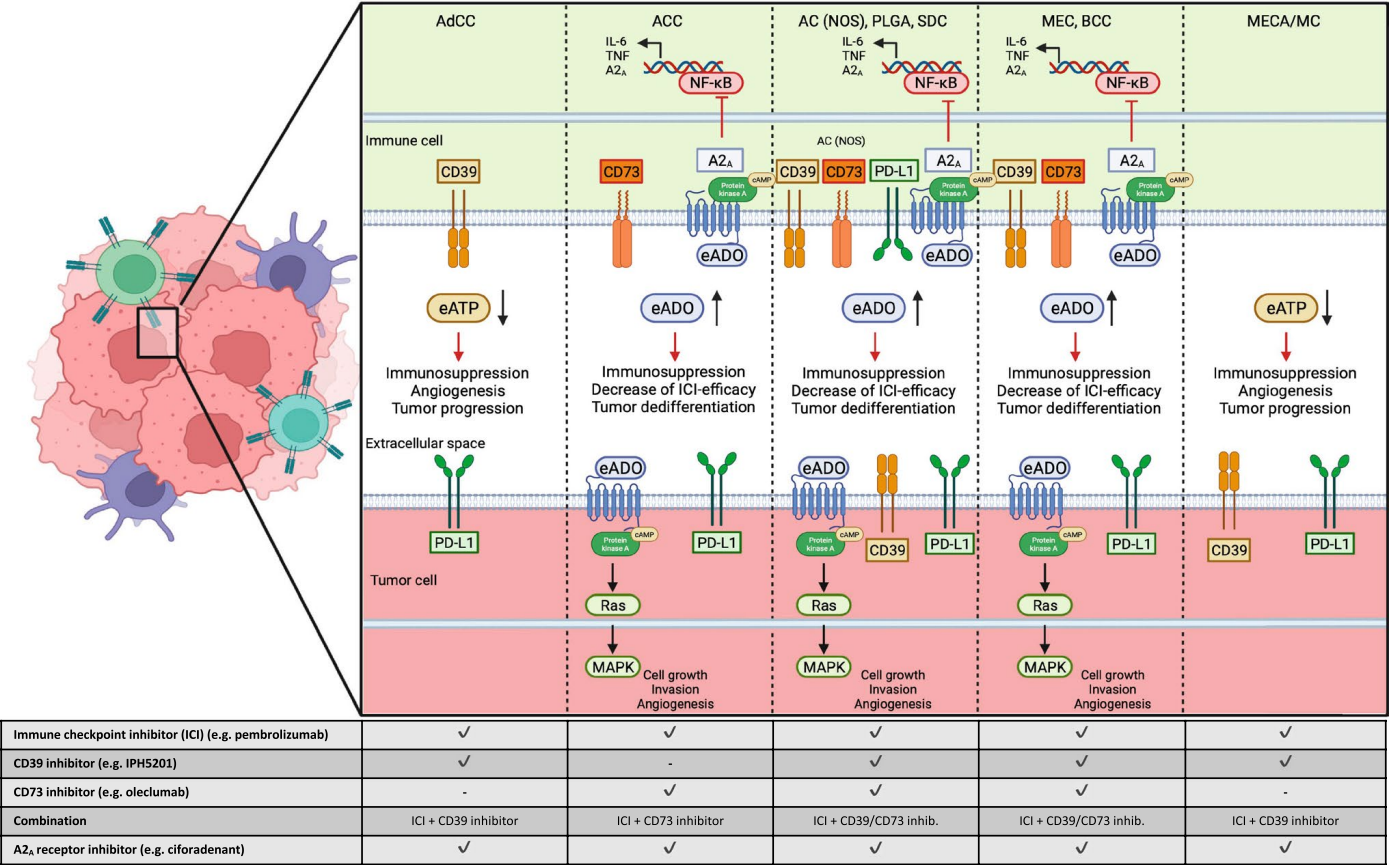
Visualization of CD39/CD73/PD-L1 expression signatures for each entity. Moreover, we provide potential therapeutic vulnerabilities based on our current results from adenosine pathway marker expression analysis (created with BioRender)

The inhibition of adenosine pathway markers, such as CD39 or CD73, poses a promising immune therapeutic strategy. Preclinical trials have already underlined the feasibility of such approaches across a variety of solid tumors (Young et al. 2016). Arising from Parkinson’s disease research, it has been shown that A2_A_ receptor antagonists (e.g., ciforadenant or taminadenant) harbor antitumor activity in cancers associated with high levels of CD39 and CD73 in the tumor microenvironment (TME) (Pinna 2014; Houthuys et al. 2019). Preliminary results from early phase trials have shown clinical efficacy for the therapeutic inhibition of CD73 by monoclonal antibodies such as oleclumab with or without the PD-L1 inhibitor durvalumab and led to PRs in heavily pretreated patients with colorectal or pancreatic carcinomas (Overman et al. 2018; Caluwe et al. 2021). The majority of studies investigating the efficacy of ICI in SGC consider pembrolizumab as the immunotherapeutic agent of choice in analogy to head and neck squamous cell carcinoma (HNSCC) (Cohen et al. 2019; Burtness et al. 2019). However, durvalumab represents the most common immunotherapeutic combination partner of adenosine pathway inhibitors in literature so far. The development of monoclonal anti-CD39 antibodies is associated with slower progress. Currently, three anti-CD39 monoclonal antibodies including IPH5201, as the most advanced candidate, are undergoing clinical testing (Perrot et al. 2019; Li et al. 2019). In SGC excluding ACC (CD73 expression on immune cells), this therapeutic strategy of targeting the adenosine pathway seems to be the most promising in light of high CD39 expression levels on both tumor and immune cells. However, CD73 displays the rate-limiting enzyme for adenosine production and is therefore predestined as a promising therapeutic target as well (Roh et al. 2020). The mechanisms of anti-adenosinergic treatment strategies for SGCs are illustrated in Fig. 7.

## Discussion

In the current study, we investigated the immuno-histochemical status of the adenosine pathway markers CD39 and CD73, two novel immunotherapeutic targets beyond the conventional anti-CTLA-4, PD-1 or PD-L1 inhibition in SGCs. In contrast to results from a previous study, we were not able to confirm frequent CD73 expression on tumor cells in neither MEC nor ACC (Ranjbar et al. 2019). In the present cohort, we detected CD73 expression exclusively on immune but not on tumor cells in ACC and MEC. Apart from this study, to the best of our knowledge, this is the first study that systematically investigated CD39 and CD73 expression patterns across the spectrum of SGCs. In a recent review article, we already underlined the urgent need of investigating the adenosine pathway markers CD39 and CD73 as potential immunotherapeutic targets in SGCs (Witte et al. 2022).

In keeping with results from lung adenocarcinoma (LUAD) and other solid tumors, we found implications for higher T cell infiltration in tumors with high CD73 expression levels on immune cells suggesting a rationale for the combination of therapeutic CD73 inhibition and ICI (Roh et al. 2020; Rocha et al. 2021; Reinhardt et al. 2017; Goswami et al. 2020). In contrast to our results, a frequent CD73 was found on tumor cells across a variety of solid tumors (Chen et al. 2019; Ma et al. 2019). However, several entities among the spectrum of aggressive solid tumors were associated with low CD73 expression levels (Eroglu et al. 2000; Durak et al. 1993; Rackley et al. 1989). Therefore, the role of CD73 expression on the prognosis of cancer patients remains insufficiently explained (Roh et al. 2020). The present results revealed a stronger rationale for the therapeutic inhibition of CD39 compared to CD73. To date, our review of literature detected no study investigating the expression levels of CD39 in any SGC-entity. The discussion about CD39 expression levels in solid tumors is less controversial. Apart from urogenital tumors, RNA sequencing showed that the majority of cancer subtypes frequently overexpress CD39 (Allard et al. 2020b). Here, we found low expression levels of CD39 on tumor cells and immune cells in MEC and AdCC, respectively. However, we detected high CD39 expression levels in the other SGCs investigated.

From the present results, three rationales for SGC treatment can be concluded that should be investigated in future studies. First, in SGC, we found frequent CD73 expression on cells of the immune infiltrate, whereas CD73 expression on tumor cells remained sparse. CD73 comes along with immunosuppressive features supporting tumor growth and decreasing ICI efficacy. Subsequently, targeting CD73 within the TME can also enhance antitumor activity within the spectrum of immunotherapeutic approaches (Perrot et al. 2019). Especially in combination with ICI, the inhibition of CD73 leads to the increase of CD8^+^ TILs and consequently support antitumor immunity (Overman et al. 2018). Apart from monoclonal antibodies, small molecules targeting CD73 (e.g., LY3475070) currently undergo clinical evaluation in patients with advanced solid tumors (NCT04148937). Additionally, bispecific antibodies (CD73/EpCAM) have been developed to inhibit the adenosine-mediated immunosuppressive activity (Ploeg et al. 2021).

Second, the list of rationales for inhibiting CD39 as the next generation’s immunotherapeutic target of choice is exhaustive. The immunosuppressive function of CD39 is reflected by the inhibition of T cell activation, T cell/NK cell effector function and NLRP3 inflammasome as well as pyroptosis. Moreover, CD39 enhances tumorigenesis and the immunosuppression by regulatory T cells (T-regs), B cells, myeloid-derived suppressor cells (MDSC) and macrophages. The enzymatic activity of CD39 restricts leukocyte migration and suppresses antigen presentation (Allard et al. 2020b). Considering the high expression levels, targeting CD39 constitutes one of the most promising immune-oncologic approaches in the majority of SGCs beyond PD-(L)1 blockage. Anti-CD39 monoclonal antibodies reduce the extracellular levels of adenosine and increase the extracellular levels of adenosine-triphosphate (eATP). Increased eATP levels can extend the activity of memory T cells by improving tumor antigen presentation of dendritic cells. In contrast to the inhibition of CD73, the activation of the eATP-dependent P2X_7_-receptor and the NLRP3 inflammasome are essential for antitumor activity of monoclonal antibodies targeting CD39 (Li et al. 2019; Yan et al. 2020).

Third, there exist several rationales for immune-therapeutic combinations. The anti-CD39-mediated activation of the NLRP3 inflammasome can augment the efficacy of CD73 blockage (Li et al. 2019). This suggests the co-inhibition of both CD39 and CD73 which is supported by preclinical trials (Li et al. 2019). As already mentioned, other preclinical and clinical studies suggested the augmented efficacy of combinations with anti-PD-L1 blockage across several solid tumors (Leone et al. 2018; Jin et al. 2010).

Limitations of the current study include the overall limited sample size and the composition of the study cohort with heterogeneous treatment characteristics as well as its retrospective design which comes along with the potential for fragmentary data. Additionally, SGC patients were not treated with inhibitors of the adenosine pathway and only a minor subset of patients received immunotherapeutic agents. Consequently, conclusions that suggest such treatments alone or the combination of adenosine pathway inhibition and ICI need to be investigated in further studies. Flow cytometry for adenosine pathway markers on immune cells would have been desirable but was beyond the scope of the present study due to missing frozen blood samples from SGC patients included in the study. Another potential shortcoming of the present study is the limited number of cases in which a comprehensive analysis of the immune infiltrate was performed. The current results might profit from the assessment of PD-1 expression levels on immune cells. Moreover, the histopathologic selectivity of AC (NOS) and adjacent entities remains an unaddressed issue to date. The comprehensive genomic analysis of these entities might lead to a reclassification of SGCs. Accordingly, biased survival measures of limited SGC subtypes by histopathologic misinterpretation cannot be ruled out.

Nevertheless, as we previously established PD-L1 scoring in SGCs, we now extend the spectrum of immuno-histochemical immune checkpoint scoring. In view of the fact that ICI alone did not provide promising results in SGCs, this is of great interest for future studies investigating the efficacy of adenosine pathway inhibitors alone or in combination with ICI. The present results underline that tumor immunity plays a crucial role in SGCs. In the era of precision oncology, the implementation of genomic insights for novel targeted therapies and, considering the importance of tumor immunity, the implementation of immunotherapeutic approaches will extend the therapeutic repertoire in aggressive SGC subtypes with poor clinical outcome, especially in AC (NOS).

## Supplementary Information

Below is the link to the electronic supplementary material.

Supplementary file 1 (DOCX 17 kb)
