# Supplementary material for: The expression of the adenosine pathway markers CD39 and CD73 in salivary gland carcinomas harbors the potential for novel immune checkpoint inhibition
Source: J Cancer Res Clin Oncol. 2022 Jul 28;149(7):3193–208. doi: 10.1007/s00432-022-04211-x (PMC10314850; doi:10.1007/s00432-022-04211-x)
Supplement: Supplementary file 1 — (DOCX 17 kb) [file 432_2022_4211_MOESM1_ESM.docx]

**Supplementary Material**

**Supplementary Table 1.** Antibodies used in the present study for immunohistochemistry.

| **Marker** | **Producer** | **Clone** | **Origin** |
| --- | --- | --- | --- |
| CD39 | Sigma-Aldrich | polyclonal | rabbit |
| CD73 | Sigma-Aldrich | polyconal | rabbit |
| PD-L1 | Roche | SP 263 | rabbit |
| CD3 | Ventana Medical Systems | 2GV6 | rabbit |
| CD20 | Cell Marque | L26 | mouse |
| CD117 | Cell Marque | YR145 | rabbit |
| P53 | Ventana Medical Systems | DO-7 | mouse |

**Supplementary Table 2.** Comprehensive analysis of the immune infiltrate of 69 patients within the present cohort.

|  | CD39^TPS^ | CD39^IPS^ | CD73^TPS^ | CD73^IPS^ | PD-L1 ^TPS^ | PD-L1 ^IPS^ | CD3 | CD20 | CD117 |
| --- | --- | --- | --- | --- | --- | --- | --- | --- | --- |
| Cohort (n=69) | 20/69 (28,9%) | 26/69 (37,7%) | 13/69 (18,8%) | 32/69 (46,4%) | 51/69 (73,9%) | 40/69 (58,0%) | 68/69 (98,6%) | 57/69 (82,6%) | 21/69 (30,4%) |
| AdCC (n=28) | 8/28 (28,6%) | 2/28 (7,1%) | 3/28 (10,7%) | 3/28 (10,7%) | 20/28 (71,4%) | 13/28 (46,4%) | 27/28 (96,4%) | 21/28 (75,0%) | 20/28 (71,4%) |
| AC, NOS (n=9) | 3/9 (33,3%) | 7/9 (77,8%) | 1/9 (11,1%) | 7/9 (77,8%) | 9/9 (100,0%) | 9/9 (100,0%) | 9/9 (100,0%) | 7/9 (77,8%) | 0/9 (0,0%) |
| ACC  (n=12) | 6/12 (50,0%) | 5/12 (41,7%) | 3/12 (25,0%) | 8/12 (66,7%) | 9/12 (75,0%) | 6/12 (50,0%) | 12/12 (100,0%) | 10/12 (83,3%) | 0/12 (0,0%) |
| MEC  (n=19) | 2/19 (10,5%) | 11/19 (57,9%) | 6/19 (31,6%) | 13/19 (68,4%) | 12/19 (63,2%) | 11/19 (57,9%) | 19/19 (100,0%) | 18/19 (94,7%) | 1/19 (5,3%) |
| SDC  (n=1) | 1/1 (100,0%) | 1/1 (100,0%) | 0/1 (0,0%) | 1/1 (100,0%) | 1/1 (100,0%) | 1/1 (100,0%) | 1/1 (100,0%) | 1/1 (100,0%) | 0/1 (0,0%) |

**Supplementary Table 3.** Overall (OS) and progression free survival for each entity included in the present study.

| **Entity** | **Overall survival (median)** | **Progression-free survival (median)** |
| --- | --- | --- |
| AdCC | 74,1 | 40,1 |
| AC, NOS | 35,5 | 13,2 |
| PLGA | 22,4 | 9,2 |
| BCC | 27,4 | 27,4 |
| ACC | 73,5 | 73,5 |
| MEC | 64,2 | 23,4 |
| MC | 23,4 | 13,2 |
| EMC | 16,2 | 16,2 |
| SDC | 8,1 | 14,2 |
